# Circulating levels of 25-hydroxyvitamin D and risk of breast cancer: a nested case-control study

**DOI:** 10.1186/bcr3390

**Published:** 2013-02-26

**Authors:** Stephanie Scarmo, Yelena Afanasyeva, Per Lenner, Karen L Koenig, Ronald L Horst, Tess V Clendenen, Alan A Arslan, Yu Chen, Göran Hallmans, Eva Lundin, Sabina Rinaldi, Paolo Toniolo, Roy E Shore, Anne Zeleniuch-Jacquotte

**Affiliations:** 1 Department of Population Health, New York University School of Medicine, 650 First Avenue, New York, NY 10016, USA; 2 Department of Oncology, Umeå University Hospital, S-90185, Umeå, Sweden; 3 New York University Cancer Institute, New York University School of Medicine, 530 First Avenue, New York, NY 10016, USA; 4 Heartland Assays Inc., 2711 South Loop Drive, Suite 4400, Ames, IA 50010, USA; 5 Department of Obstetrics and Gynecology, New York University School of Medicine, 550 First Avenue, New York, NY 10016, USA; 6 Department of Public Health and Clinical Medicine/Nutritional Research, Umeå University, SE-901 87, Umeå, Sweden; 7 Department of Medical Biosciences/Pathology, Umeå University, SE-901 85, Umeå, Sweden; 8 International Agency for Research on Cancer, 150, Course Albert Thomas, 69372 Lyon Cedex 08, France; 9 Radiation Effects Research Foundation, 5-2 Hijiyama Park, Minami-ku, Hiroshima, 732-0815, Japan

## Abstract

**Introduction:**

Experimental evidence suggests a protective role for circulating 25-hydroxyvitamin D (25(OH)D) in breast cancer development, but the results of epidemiological studies have been inconsistent.

**Methods:**

We conducted a case-control study nested within two prospective cohorts, the New York University Women's Health Study and the Northern Sweden Mammary Screening Cohort. Blood samples were collected at enrollment, and women were followed up for breast cancer ascertainment. In total, 1,585 incident breast cancer cases were individually-matched to 2,940 controls. Of these subjects, 678 cases and 1,208 controls contributed two repeat blood samples, at least one year apart. Circulating levels of 25(OH)D were measured, and multivariate odds ratios (ORs) and 95% confidence intervals (CIs) were calculated using conditional logistic regression.

**Results:**

No association was observed between circulating levels of 25(OH)D and overall breast cancer risk (multivariate-adjusted model OR = 0.94, 95% CI = 0.76-1.16 for the highest vs. lowest quintile, *p*_trend _= 0.30). The temporal reliability of 25(OH)D measured in repeat blood samples was high (intraclass correlation coefficients for season-adjusted 25(OH)D > 0.70). An inverse association between 25(OH)D levels and breast cancer risk was observed among women who were ≤ 45 years of age (OR_Q5-Q1 _= 0.48, 95% CI = 0.30-0.79, *p*_trend _= 0.01) or premenopausal at enrollment (OR_Q5-Q1 _= 0.67, 95% CI = 0.48-0.92, *p*_trend _= 0.03).

**Conclusions:**

Circulating 25(OH)D levels were not associated with breast cancer risk overall, although we could not exclude the possibility of a protective effect in younger women. Recommendations regarding vitamin D supplementation should be based on considerations other than breast cancer prevention.

## Introduction

Experimental studies support a role for vitamin D in reducing the risk of breast cancer [1,2]. Vitamin D, which is obtained from both dietary sources (food and supplements) and exposure to type B ultraviolet radiation, undergoes two hydroxylation steps before becoming biologically active [3]. 25-hydroxyvitamin D [25(OH)D], produced in the liver from the first hydroxylation, is the precursor of the biologically active form, 1,25(OH)_2_D, which is produced in the kidney as well as in target tissues, including the breast [4]. Circulating 25(OH)D is considered the best marker of vitamin D status because it reflects vitamin D obtained from both diet/supplements and sun exposure [5] and has a longer half-life than 1,25(OH)_2_D [6]. Vitamin D acts by binding to the vitamin D receptor (VDR), which is expressed in mammary tissue. The VDR controls the expression of genes regulating cell proliferation, differentiation, and apoptosis [1,7].

The results of epidemiologic studies examining the association between circulating 25(OH)D levels and breast cancer risk have been inconsistent. Seven prospective studies reported no association overall [8-14], whereas three reported a significant or marginally significant inverse association [15-17]. Some significant findings emerged from the results of subgroup analyses, although the subgroups of women for whom these associations were observed were not consistent across studies. The Nurses' Health Study observed a stronger protective effect of plasma 25(OH)D on breast cancer risk for women who were 60 years old or older [16], whereas the French E3N cohort observed a stronger effect in women who were less than 53 years old at enrollment [15]. In the Nurses' Health Study II, which consists primarily of pre-menopausal women, no association was observed overall between plasma 25(OH)D levels and breast cancer risk, and a positive, rather than an inverse, association was observed among women who were overweight or obese, the latter of which is defined as a body mass index (BMI) of at least 30 kg/m^2 ^[13].

Recent reviews have concluded that there is insufficient evidence to recommend vitamin D supplementation for the prevention of breast cancer but that additional research in humans is needed [3,18,19]. The purpose of our study was to examine the association between pre-diagnostic circulating levels of circulating 25(OH)D and breast cancer risk in a case-control study nested within two prospective cohorts: the New York University Women's Health Study (NYUWHS) and the Northern Sweden Mammary Screening Cohort (NSMSC). A unique feature of this study, the largest prospective study to date, was the availability of two 25(OH)D measurements from blood samples donated a minimum of one year apart for a large proportion of the study subjects, allowing us to estimate with good precision the temporal reliability of 25(OH)D, and factors that affected it, in the two cohorts.

## Materials and methods

### Study population

Descriptions of the NYUWHS and the NSMSC have been provided previously [20,21]. Briefly, the NYUWHS cohort enrolled 14,274 healthy women (34 to 65 years old) at a mammography screening clinic in New York City between 1985 and 1991, and the NSMSC enrolled, between 1995 and 2006, approximately 28,800 women (40 to 69 years old) who are participating in a population-based breast cancer screening program in Västerbotten County, Sweden. After written informed consent was obtained from all study participants, information on demographic and anthropometric variables, medical and reproductive history, family history of breast cancer, and lifestyle factors, including diet, was collected through baseline or subsequent questionnaires or both. Venous blood was collected at enrollment, processed according to standard procedures by the respective cohorts (serum for NYUWHS and plasma for NSMSC), and stored at -80°C. Additional blood samples were collected from women who returned for screening. This study was approved by the Institutional Review Board of the New York University School of Medicine, the Regional Ethics Committee of the University of Umeå, Sweden, and the Swedish Data Inspection Board.

### Case ascertainment and control selection

For the NYUWHS, incident cases of invasive breast cancer were identified by mailed questionnaires or follow-up telephone interviews every 2 to 4 years after 1991, supplemented by linkages to state cancer registries in New York, New Jersey, and Florida and the US National Death Index. Medical records were reviewed to confirm self-reported cases. Using a capture-recapture analysis, we estimated that combining active and cancer registry-based follow-up resulted in a breast cancer ascertainment rate of 95% [22]. For the NSMSC, annual linkages to the Swedish National Cancer Registry were used to identify incident cases of breast cancer in the cohort. As of 1 January 2007 for the NYUWHS and 1 January 2010 for the NSMSC, a total of 1,645 incident cases of invasive breast cancer (909 in the NYUWHS and 736 in the NSMSC) had been identified. In the NYUWHS, 16 cases (3%) were excluded because they had a low serum balance. In the NSMSC, 44 cases (6%) were excluded for the following reasons: 26 had low plasma balance, 13 had their plasma reserved because of a subsequent diagnosis of a rarer cancer or other disease, 3 had insufficient volume for laboratory measurement, and 2 had both matched controls excluded for one of the reasons above. The present study included a total of 1,585 incident breast cancer cases (893 from the NYUWHS and 692 from the NSMSC).

Each case was matched to two controls who were selected from the respective cohort by using incidence-density sampling. Matching factors included age at enrollment in the study (± 6 months), date of enrollment/first blood donation (NYUWHS: ± 3 months; NSMSC: ± 1 month), and number (1, 2+) and dates of subsequent blood donations. For the NYUWHS, matching factors also included menopausal status (pre- or post-menopausal) at enrollment and race/ethnicity (Caucasian, African-American, other, or unknown). The vast majority of women in the NSMSC were Caucasian. Initially, women were not matched on menopausal status in this cohort; however, 88% of the cases had at least one control matching on this factor (pre- and peri-menopausal combined or post-menopausal). In total, 2,940 controls were included in the final analysis (1,642 in NYUWHS and 1,298 in NSMSC).

For 678 matched sets (413 in NYUWHS and 265 in NSMSC), two blood samples were analyzed for 25(OH)D. For participants who had donated more than two blood samples, the first and last samples collected before the relevant case's diagnosis were selected.

### Laboratory methods

Circulating 25(OH)D was measured by Heartland Assays, Inc. (Ames, IA, USA) by using a direct competitive chemiluminescence immunoassay by using the DiaSorin LIAISON platform (DiaSorin, Inc., Stillwater, MN, USA) [23]. The assay, which is appropriate for either serum or plasma, is co-specific for 25-hydroxyvitamin D_3 _and 25-hydroxyvitamin D_2_. All samples, including repeat samples, from a case and her matched controls were analyzed together in the same laboratory batch to minimize laboratory variability. Laboratory personnel were blinded to case-control status of the study samples. Samples from quality-control pools (6% of total samples) were masked and inserted randomly in the batches. The intra- and inter-batch coefficients of variation (CVs) were 9.5% and 11.4%, respectively, for NYUWHS and 7.4% and 9.0%, respectively, for NSMSC.

Estrone was measured by double-antibody radioimmunoassay with reagents from Diagnostic System Laboratories (Webster, TX, USA) at the Laboratory for Hormone Analyses at the International Agency for Research on Cancer, France, for post-menopausal women who were not using hormone replacement therapy (HRT). Intra- and inter-batch CVs were 6.7% and 12.6%, respectively [24,25].

### Statistical analysis

We examined the temporal reliability of circulating 25(OH)D by using the intraclass correlation coefficient (ICC). In addition to calculating the overall ICC, we calculated ICCs according to time (years) between sample donations and according to season, for each cohort separately.

Conditional logistic regression was used to estimate odds ratios (ORs) and 95% confidence intervals (CIs) for the associations of subject characteristics and circulating 25(OH)D with risk of breast cancer. We conducted analyses separately for each cohort as well as combining them. Because there was no evidence of cohort heterogeneity, most results are presented for the combined cohorts. 25(OH)D concentrations were log_2_-transformed to reduce departure from the normal distribution and were included in the model in one of three ways. First, we computed season-adjusted residuals to take into account the known variations of 25(OH)D with season [3]. For each 25(OH)D measurement, the residual was the difference between the observed 25(OH)D value and the value predicted for this day of the year, which was obtained by using a non-parametric local regression (Proc LOESS; SAS Institute Inc., Cary, NC, USA) with 25(OH)D as the dependent variable and day of the year of blood donation as the independent variable [26]. This regression model was run separately for each cohort by using all available 25(OH)D measurements (that is, including repeat samples). We conducted analyses by using cohort-specific season-adjusted quintiles based on the distribution of the residuals in controls. Second, we ran analyses with season-adjusted residuals on the continuous scale. All analyses based on residuals were conducted by using first (that is, baseline) samples only. Finally, we examined the association of circulating 25(OH)D with breast cancer risk by using pre-specified categories of 25(OH)D levels, which were defined by using cut-points recommended by the Institute of Medicine: less than 50 (inadequate), 50 to 74 (adequate), and at least 75 nmol/L (adequate to high) [3]. These analyses were conducted separately for 'winter' and 'summer', which were defined by examining the unadjusted 25(OH)D levels in controls within each cohort. Winter included the months of January to April, when mean levels were low (48.1 to 51.2 nmol/L), and summer included the months of July to September, when mean levels were high (62.0 to 68.0 nmol/L). There was little variation in mean level from month to month within each of these two seasons. Subjects who had measurements in both winter and summer were included in both season-specific analyses to increase the sample size. Because there was no difference in the main study findings between conditional logistic regression models and unconditional models adjusting for the matching factors and because using conditional regression resulted in the loss of matched sets with samples collected in different seasons for the case and her controls, unconditional logistic regression was used for the season-specific analyses. In analyses by quintiles and pre-specified categories, tests for trend were performed by using an ordered categorical variable. Tests for heterogeneity were carried out by comparing models that included interaction terms to models that excluded them or by using Cochran's Q statistic.

In each of the two cohorts, multivariate linear regression analyses were conducted among the controls to explore associations of potential confounders with 25(OH)D. BMI was found to be a negative predictor of 25(OH)D, whereas multivitamin supplement use and past use of HRT were positive predictors in both cohorts. Caucasian race and physical activity were also positive predictors of 25(OH)D in the NYUWHS, and alcohol consumption was a negative predictor in the NSMSC. Covariates in the final multivariate models included the following known breast cancer risk factors: age at menarche (continuous), age at first birth/parity (not more than 20, 21 to 15, 26 to 30, more than 30 years, nulliparous), family history of breast cancer (no or yes), BMI (continuous), past HRT use (never or ever), and alcohol consumption. It is debatable whether to control for outdoor physical activity and multivitamin use, which have been associated with higher levels of circulating 25(OH)D [27,28] since these variables may influence breast cancer risk through their effect on 25(OH)D levels. However, these factors may affect breast cancer risk through other mechanisms [29], in which case they could act as possible confounders in analyses of 25(OH)D and breast cancer risk. Therefore, in addition to presenting models adjusting for the factors listed above, we present results adjusting for physical activity and multivitamin use (no or yes). In the NYUWHS, physical activity was expressed as metabolic equivalent of task-hours per week (MET-hours/week) from walking and vigorous exercise, and women were classified into tertiles. In the NSMSC, women were classified as inactive, moderately active, or active by combining data on physical activity at work and frequency of walking, biking, and exercising. Baseline data were used for all variables except HRT in the NYUWHS, which represented use up to the date of diagnosis (or index date for controls).

We performed multiple imputation by using fully conditional specification [30] for the following covariates with missing data: alcohol consumption (23%), physical activity (23%), multivitamin use (18%), HRT use (6%), age at menarche, parity, age at first full-term pregnancy, and BMI (all with not more than 2% missing data). We compared analyses including all subjects and imputed data for covariates to analyses including only subjects with no missing data (complete case method). Because results from both analyses were similar, we present only the analyses that included all subjects and imputed data.

We conducted stratified analyses by using conditional logistic regression for the following variables: age at enrollment, lag-time between blood donation and diagnosis, and estrogen receptor (ER) status. In order not to lose the matched sets in which a case and her controls were discordant, unconditional logistic regression, adjusted for cohort and age at blood sampling, was performed for the following variables: menopausal status, BMI, circulating estrone levels (for post-menopausal women only), and insulin-like growth factor 1 (IGF-I) levels. Tertiles were used for the IGF-1 analysis because of the limited number of women for whom IGF-1 had been measured (193 cases and 269 controls from the NYUWHS only). Finally, we performed an analysis limited to Caucasians (90% of subjects). All significance testing was two-sided, and a *P *value of less than 0.05 was considered statistically significant.

## Results

Descriptive statistics for the breast cancer cases and their matched controls are presented in Table 1. Mean age at enrollment was 54 years for both cases and controls. Cases were diagnosed an average of 8.7 years after blood donation. Established risk factors for breast cancer - including younger age at menarche, nulliparity, older age at first full-term pregnancy, and having a first-degree family history of breast cancer - occurred more commonly in cases. Cases were more likely to report having used HRT. BMI was significantly different between cases and controls in post-menopausal women, among whom a greater proportion of cases were overweight and obese. Among the 77% of cases for which receptor status was known, 78% of tumors were ER-positive.

**Table 1:** Characteristics of breast cancer cases and matched controls

Characteristic	Case subjects Number (percentage)	Control subjects Number (percentage)	*P *value^a^
Age at enrollment, years			
≤ 45	301 (19%)	575 (20%)	
46-54	557 (35%)	1,005 (34%)	Matched
≥ 55	727 (46%)	1,360 (46%)	

Age at diagnosis, years			
≤ 55	393 (25%)		
56-64	511 (32%)		
≥ 65	681 (43%)		

Race			
Caucasian	1,370 (90%)	2,484 (90%)	
African-American	94 (6%)	163 (6%)	Matched
Other	51 (4%)	118 (4%)	
Missing	70	175	

Education			
Some high school or less	288 (25%)	582 (26%)	
Completed high school	415 (36%)	820 (37%)	0.09
College or higher	439 (38%)	801 (36%)	
Missing	443	737	

Menopausal status at enrollment			
Pre- or peri-menopausal	637 (40%)	1,134 (39%)	Matched^b^
Post-menopausal	948 (60%)	1,806 (61%)	

Age at menarche, years			
< 12	285 (18%)	477 (17%)	
12	397 (26%)	670 (23%)	
13	439 (28%)	803 (28%)	0.005
> 13	438 (28%)	926 (32%)	
Missing	26	64	

Nulliparous	327 (21%)	530 (18%)	0.029
Missing	46	63	

Age at first full-term pregnancy, years			
≤ 20	171 (14%)	409 (18%)	
21-25	523 (44%)	1,016 (44%)	
26-30	318 (27%)	596 (26%)	0.006
> 30	172 (15%)	281 (12%)	
Missing	28	45	

Ever user of oral contraceptives	677 (46%)	1219 (45%)	0.45
Missing	121	209	

Ever user of hormone replacement therapy	445 (30%)	685 (25%)	< 0.001
Missing	99	176	

First-degree family history of breast cancer	299 (19%)	437 (15%)	0.0005

Body mass index, kg/m^2^			

Pre- and peri-menopausal			
< 20.0	53 (8%)	104 (9%)	
20.0-24.9	339 (54%)	614 (55%)	0.24
≥ 25.0	237 (38%)	409 (36%)	
Missing	8	7	

Post-menopausal			
< 20.0	31 (3%)	78 (4%)	
20.0-24.9	396 (43%)	848 (48%)	
≥ 25.0	498 (54%)	835 (48%)	0.0009
Missing	23	45	

Multivitamin user	515 (41%)	956 (39%)	0.49
Missing	332	509	

Ever smoker	642 (50%)	1,264 (52%)	0.52
Missing	299	524	

Alcohol, drinks per day			
0	501 (43%)	928 (41%)	
< 1	552 (47%)	1,161 (51%)	
≥ 1	123 (10%)	187 (8%)	0.80
Missing	409	664	

Physical activity			

NYUWHS, MET-hours per week			
< 5.5	249 (31%)	412 (28%)	
5.5-21.5	271 (34%)	506 (35%)	0.12
≥ 21.5	274 (35%)	548 (37%)	
Missing	99	176	

Mammary Screening Cohort, activity level			
Inactive	55 (14%)	107 (13%)	
Moderately active	145 (37%)	305 (37%)	
Active	192 (49%)	408 (50%)	0.84
Missing	300	478	

Dietary vitamin D in IU/day, median (10th-90th percentile)	141 (55, 272)	145 (55, 275)	0.60
Missing	340	526	

Circulating 25(OH)D in nmol/L, median (10-90th percentile)	53.0 (31.2, 81.0)	54.2 (30.7, 82.6)	0.27

^a^*P *value from conditional logistic regression (*P *value from trend test for ordered categories). ^b^Menopausal status (pre- or post-menopausal) was a matching factor for all sets in the New York University Women's Health Study (NYUWHS). For Northern Sweden Mammary Screening Cohort (NSMSC), women were not initially matched on this factor, and 88% of the cases had at least one control matched on menopausal status (pre- and peri-menopausal combined or post-menopausal). 25(OH)D, 25-hydroxyvitamin D; MET, metabolic equivalent of task.

For women who donated more than one blood sample, the average time between sample donations was 2.1 years in the NYUWHS and 4.4 years in the NSMSC. Overall, the temporal reliability of 25(OH)D was good (ICC = 0.65, 95% CI 0.61 to 0.69 for NSMSC and 0.78, 95% CI 0.76 to 0.80 for NYUWHS) and improved for NSMSC when season-adjusted residuals were used (ICC = 0.71, 95% CI 0.67 to 0.74) (Table 2). The ICC for the NYUWHS was not changed by seasonal adjustment, because women in the NYUWHS generally returned to the screening center and donated a blood sample at the same time each year. We observed that the ICC decreased as time increased between sample donations, although this trend did not appear to extend beyond the first 8 years. For the NSMSC, the season-adjusted ICCs were 0.56 (95% CI = 0.42 to 0.68) for samples collected 5 to 8 years apart (*n *= 113 subjects) and 0.63 (95% CI = 0.51 to 0.74) for samples collected between 8 and 11.7 years apart (*n *= 106 subjects). For both cohorts, the ICC was substantially lower when one sample was donated in the winter and the other one in the summer months (ICC = 0.47, 95% CI = 0.29 to 0.61 for NSMSC, *n *= 92; ICC = 0.66, 95% CI = 0.50 to 0.77 for the NYUWHS, *n *= 68).

**Table 2:** Intraclass correlation coefficients by time between sample donation (years) and by season (NSMSC and NYUWHS)

	NSMSC		NYUWHS	
	
	Number of subjects	ICC (95% CI)	Number of subjects	ICC (95% CI)
All^a^	700	0.65 (0.61, 0.69)	1,168	0.78 (0.76, 0.80)

All^b^	700	0.71 (0.67, 0.74)	1,168	0.78 (0.76, 0.80)

Time between samples^b^, years				

≤ 1			393	0.80 (0.76, 0.83)
> 1-2	146	0.79 (0.71, 0.84)	325	0.81 (0.77, 0.84)
> 2-3	139	0.81 (0.75, 0.86)	208	0.80 (0.75, 0.84)
> 3-5	196	0.71 (0.63, 0.73)	214	0.74 (0.67, 0.79)
> 5-8	113	0.56 (0.42, 0.68)		
> 8-11.7	106	0.63 (0.51, 0.74)		

Seasons^a^				
Both samples in winter	148	0.74 (0.66, 0.81)	266	0.78 (0.72, 0.82)
Both samples in summer	53	0.79 (0.67, 0.88)	210	0.81 (0.76, 0.85)
One sample in winter, one in summer	92	0.47 (0.29, 0.61)	68	0.66 (0.50, 0.77)

^a^Log_2_-transformed 25(OH)D values. ^b^Season-adjusted 25(OH)D residual values. 25(OH)D, 25-hydroxyvitamin D; CI, confidence interval; ICC, intraclass correlation coefficient; NSMSC, Northern Sweden Mammary Screening Cohort; NYUWHS, New York University Women's Health Study.

Table 3 reports ORs and 95% CIs for breast cancer risk according to season-adjusted quintiles of 25(OH)D. There was no association between circulating vitamin D and breast cancer risk overall (adjusted model OR_Q5-Q1 _= 0.94, 95% CI = 0.76 to 1.16, *P*_trend _= 0.30) or within either cohort. Results with 25(OH)D on the continuous scale were similar. Adjusting for physical activity and multivitamin use, in addition to the other confounders, did not materially affect the results (OR_Q5-Q1 _= 0.94, 95% CI = 0.76 to 1.16, *P*_trend _= 0.27).

**Table 3:** Odds ratios and 95% confidence intervals for breast cancer risk according to season-adjusted circulating levels of 25(OH)D (by quintiles and as a continuous variable)

	Quintiles						Continuous	
	
	1	2	3	4	5	*P*_trend_	OR (95% CI)^a^	*P *value
Both cohorts								
Cases/controls	311/589	362/588	309/588	315/588	288/587			
Unadjusted model	1.0 (ref)	1.18 (0.97, 1.43)	0.99 (0.81, 1.21)	1.02 (0.84, 1.24)	0.93 (0.76, 1.14)	0.18	0.94 (0.84, 1.06)	0.31
Adjusted model^b^	1.0 (ref)	1.16 (0.95, 1.42)	1.00 (0.81, 1.22)	1.04 (0.85, 1.27)	0.94 (0.76, 1.16)	0.30	0.95 (0.84, 1.08)	0.44
Adjusted model^c^	1.0 (ref)	1.16 (0.95, 1.42)	0.99 (0.81, 1.21)	1.03 (0.84, 1.27)	0.94 (0.76, 1.16)	0.27	0.95 (0.84, 1.07)	0.40

NYUWHS								
Cases/controls	191/329	190/328	176/329	170/328	166/328			
Unadjusted model	1.0 (ref)	0.99 (0.76, 1.29)	0.92 (0.71, 1.20)	0.89 (0.69, 1.16)	0.86 (0.66, 1.12)	0.18	0.91 (0.79, 1.05)	0.21
Adjusted model^b^	1.0 (ref)	0.98 (0.75, 1.28)	0.94 (0.72, 1.23)	0.92 (0.70, 1.21)	0.88 (0.67, 1.16)	0.31	0.93 (0.80, 1.08)	0.34
Adjusted model^c^	1.0 (ref)	1.00 (0.76, 1.31)	0.95 (0.73, 1.24)	0.93 (0.71, 1.22)	0.90 (0.68, 1.19)	0.37	0.94 (0.81, 1.09)	0.42

NSMSC								
Cases/controls	120/260	172/260	133/259	145/260	122/259			
Unadjusted model	1.0 (ref)	1.47 (1.09, 1.97)	1.10 (0.81, 1.49)	1.22 (0.90, 1.64)	1.03 (0.75, 1.40)	0.62	1.00 (0.82, 1.23)	0.99
Adjusted model^b^	1.0 (ref)	1.48 (1.08, 2.01)	1.09 (0.79, 1.49)	1.26 (0.92, 1.72)	1.05 (0.76, 1.46)	0.74	1.02 (0.82, 1.26)	0.89
Adjusted model^c^	1.0 (ref)	1.46 (1.07, 1.99)	1.08 (0.79, 1.48)	1.24 (0.91, 1.70)	1.04 (0.75, 1.45)	0.71	1.01 (0.81, 1.26)	0.94

^a^Odds ratio (OR) for a doubling in 25(OH)D level. ^b^Adjusted for age at menarche (continuous), age at first birth/parity (≤ 20, 21-25, 26-30, > 30 years, nulliparous), family history of breast cancer (no or yes), body mass index (continuous), hormone replacement therapy use (never or ever), and alcohol consumption (continuous). ^c^Adjusted for physical activity and multivitamin use in addition to variables in footnote a. 25(OH)D, 25-hydroxyvitamin D; CI, confidence interval; NSMSC, Northern Sweden Mammary Screening Cohort; NYUWHS, New York University Women's Health Study; ref, reference.

In analyses using pre-specified categories of circulating 25(OH)D (not adjusted for season), we observed no association with breast cancer risk for samples taken either in the winter, when more than half of the subjects had levels below 50 nmol/L, or in the summer, when more of the subjects had levels more than 75 nmol/L (Table 4). A suggestive protective effect was observed for women in the NYUWHS who donated blood in the summer months (OR = 0.69, 95% CI = 0.45 to 1.07, *P*_trend _= 0.10 for concentrations of at least 75 versus less than 50 nmol/L), but no such association was observed in the NSMSC.

**Table 4:** Odds ratios and 95% confidence intervals for breast cancer risk according to pre-specified categories of circulating 25(OH)D concentration by season^a^

		Pre-specified categories, nmol/L				Continuous^b^	*P *value
				
		< 50	50-74	75+	*P*_trend_		
Winter (Jan., Feb., March, April)							

Both cohorts^c^	Cases/controls	391/704	244/472	65/116			
	OR (95% CI)	1.0 (ref)	0.94 (0.77, 1.15)	1.06 (0.76, 1.48)	0.95	0.97 (0.82, 1.15)	0.74

NYUWHS	Cases/controls	193/331	105/217	35/65			
	OR (95% CI)	1.0 (ref)	0.82 (0.61, 1.10)	0.91 (0.58, 1.44)	0.35	0.90 (0.73, 1.13)	0.37

NSMSC	Cases/controls	198/373	139/255	30/51			
	OR (95% CI)	1.0 (ref)	1.10 (0.83, 1.46)	1.25 (0.76, 2.06)	0.34	1.11 (0.85, 1.47)	0.44

Summer (July, Aug., Sept.)							

Both cohorts^c^	Cases/controls	113/186	217/348	99/212			
	OR (95% CI)	1.0 (ref)	1.02 (0.75, 1.38)	0.76 (0.53, 1.09)	0.14	0.82 (0.64, 1.05)	0.12

NYUWHS	Cases/controls	77/121	125/190	67/148			
	OR (95% CI)	1.0 (ref)	1.07 (0.73, 1.56)	0.69 (0.45, 1.07)	0.10	0.80 (0.60, 1.07)	0.14

NSMSC	Cases/controls	36/65	92/158	32/64			
	R (95% CI)	1.0 (ref)	0.99 (0.59, 1.67)	0.89 (0.46, 1.70)	0.72	0.88 (0.52, 1.47)	0.61

^a^Adjusted for age at menarche (continuous), family history of breast cancer (no or yes), age at first birth/parity (≤ 20, 21-25, 26-30, > 30 years, nulliparous), body mass index (continuous), hormone replacement therapy use (never or ever), and alcohol consumption (continuous). ^b^Odds ratio (OR) for a doubling in 25(OH)D level. ^c^Adjusted for cohort in addition to all factors in footnote a. 25(OH)D, 25-hydroxyvitamin D; CI, confidence interval; NSMSC, Northern Sweden Mammary Screening Cohort; NYUWHS, New York University Women's Health Study; ref, reference.

Table 5 shows the results of subgroup analyses. Higher circulating 25(OH)D was associated with a decreased risk of breast cancer among women who were pre-menopausal at blood donation (OR_Q5-Q1 _= 0.67, 95% CI = 0.48 to 0.92, *P*_trend _= 0.03) but not among those who were post-menopausal (OR_Q5-Q1 _= 1.21, 95% CI = 0.92 to 1.58, *P*_trend _= 0.67, *P*_interaction _= 0.05). A similar protective effect was observed for women who were not more than 45 years old at blood donation (OR_Q5-Q1 _= 0.48, 95% CI = 0.30 to 0.79, *P*_trend _= 0.01, *P*_interaction _= 0.08). There was no evidence of effect modification by ER status of the tumor, lag-time between blood sampling and diagnosis, BMI, or circulating estrone levels. Results of the analysis limited to Caucasians were similar to those of the analysis that included all subjects.

**Table 5:** Stratified odds ratios and 95% confidence intervals for breast cancer risk according to quintiles of season-adjusted residual values of circulating 25(OH)D concentration at enrollment

		Quintiles						
	
		1	2	3	4	5	*P*_trend_	*P*_het_^a^
Caucasians^b^								
	Cases/controls	209/399	298/449	270/469	282/473	244/471	0.46	0.31
	OR (95% CI)	1.00	1.28 (1.01, 1.62)	1.10 (0.87, 1.38)	1.17 (0.92, 1.48)	0.97 (0.76, 1.24)		

Age at enrollment, years^b^								
≤ 45	Cases/controls	72/101	54/103	62/103	61/110	52/136		
	OR (95% CI)	1.00	0.67 (0.42, 1.07)	0.78 (0.49, 1.25)	0.73 (0.46, 1.14)	0.48 (0.30, 0.79)	0.01	0.08
45-54	Cases/controls	118/220	116/220	97/201	117/209	109/186		
	OR (95% CI)	1.00	0.92 (0.65, 1.29)	0.82 (0.58, 1.17)	0.99 (0.70, 1.40)	1.04 (0.73, 1.48)	0.73	
≥ 55	Cases/controls	121/268	192/265	150/284	137/269	127/265		
	OR (95% CI)	1.00	1.76 (1.30, 2.38)	1.28 (0.94, 1.73)	1.28 (0.94, 1.75)	1.20 (0.87, 1.66)	0.96	

Lag-time to diagnosis^b^								
≤ 8 years	Cases/controls	123/261	174/240	144/258	140/253	141/252		
	OR (95% CI)	1.00	1.62 (1.19, 2.22)	1.23 (0.90, 1.69)	1.26 (0.92, 1.73)	1.18 (0.85, 1.63)	0.99	0.30
> 8 years	Cases/controls	188/328	188/348	165/330	175/335	157/335		
	OR (95% CI)	1.00	0.92 (0.71, 1.20)	0.86 (0.66, 1.13)	0.91 (0.70, 1.19)	0.82 (0.62, 1.08)	0.20	

ER status^b^								
ER^+^	Cases/controls	173/355	219/347	178/358	200/355	78/352		
	OR (95% CI)	1.00	1.31 (1.00, 1.70)	1.04 (0.80, 1.35)	1.22 (0.93, 1.58)	1.10 (0.83, 1.44)	0.79	0.79
ER^-^	Cases/controls	53/99	60/99	56/111	49/93	54/95		
	OR (95% CI)	1.00	1.03 (0.63, 1.70)	0.99 (0.60, 1.64)	1.00 (0.61, 1.66)	1.08 (0.64, 1.85)	0.85	

Menopausal status^c^								
Pre-menopausal	Cases/controls	150/229	128/228	122/218	123/221	114/238		
	OR (95% CI)	1.00	0.80 (0.59, 1.09)	0.79 (0.58, 1.08)	0.80 (0.58, 1.09)	0.67 (0.48, 0.92)	0.03	0.05
Post-menopausal	Cases/controls	161/360	234/360	187/370	192/367	174/349		
	OR (95% CI)	1.00	1.48 (1.15, 1.90)	1.16 (0.89, 1.50)	1.22 (0.94, 1.58)	1.21 (0.92, 1.58)	0.67	

BMI^c^, kg/m^2^								
< 25	Cases/controls	115/245	153/312	164/323	195/361	192/403		
	OR (95% CI)	1.00	1.01 (0.75, 1.36)	1.05 (0.78, 1.40)	1.10 (0.83, 1.47)	0.97 (0.73, 1.29)	0.99	0.56
25+	Cases/controls	187/331	201/267	142/260	115/215	90/171		
	OR (95% CI)	1.00	1.31 (1.01, 1.70)	0.93 (0.70, 1.22)	0.91 (0.68, 1.22)	0.93 (0.68, 1.27)	0.16	

Estrone, pg/mL^c, d^								
< 23.25	Cases/controls	32/90	42/85	40/94	44/97	33/102		
	OR (95% CI)	1.00	1.38 (0.79, 2.41)	1.19 (0.68, 2.09)	1.36 (0.78, 2.36)	0.95 (0.53, 1.70)	0.83	0.41
23.25-32.45	Cases/controls	43/83	59/82	48/91	53/81	47/74		
	OR (95% CI)	1.00	1.40 (0.84, 2.33)	1.03 (0.61, 1.73)	1.33 (0.78, 2.25)	1.29 (0.75, 2.22)	0.49	
> 32.45	Cases/controls	48/83	80/94	56/80	37/82	41/52		
	OR (95% CI)	1.00	1.70 (1.05, 2.76)	1.36 (0.81, 2.27)	0.87 (0.50, 1.50)	1.66 (0.94, 2.95)	0.82	

^a^Cochran's Q statistic was used to test for heterogeneity according to lag-time and estrogen receptor (ER) status. ^b^Adjusted for age at menarche (continuous), family history of breast cancer (yes or no), age at first birth/parity (≤ 20, 21-25, 26-30, > 30 years, nulliparous), body mass index (BMI) (continuous), hormone replacement therapy use (ever or never), and alcohol consumption (continuous). ^c^Unconditional logistic regression analyses adjusted for cohort and age at sampling in addition to all factors in footnote b. ^d^Measured in post-menopausal women only. 25(OH)D, 25-hydroxyvitamin D; CI, confidence interval; OR, odds ratio.

When we examined the association of 25(OH)D with breast cancer risk by IGF-1 levels at baseline, the ORs for the third tertile were 0.62 (95% CI = 0.30 to 1.28) in the below-median stratum and 0.79 (95% CI = 0.39 to 1.62) in the above-median stratum. The test for interaction between IGF-1 and 25(OH)D on the continuous scale was not statistically significant (*P *= 0.61).

## Discussion

In this case-control study nested within two cohorts, we did not observe an association between circulating 25(OH)D and breast cancer risk overall. We observed an inverse association between 25(OH)D and breast cancer risk in the subgroups of women who were not more than 45 years old or pre-menopausal at blood donation, although the test for interaction was significant only for menopausal status. Because of substantial overlap, we were not able to sort out whether younger age or pre-menopausal status was driving the association.

Epidemiologic studies on vitamin D and breast cancer risk have been reviewed recently [31,32]. Traditional case-control studies [33-39] with blood samples collected after breast cancer diagnosis have found inverse associations between circulating 25(OH)D and breast cancer risk. However, changes in lifestyle, particularly physical activity, following breast cancer diagnosis and treatment may affect circulating 25(OH)D, and associations observed in these studies therefore may not reflect pre-diagnosis associations [32]. Among the 10 prospective studies published to date, eight reported no association overall [8-14], one reported a marginally significant inverse association [16], and one reported a significant inverse association [15].

In regard to younger and pre-menopausal women, data from prospective studies are more limited since eligibility in some of the largest studies was restricted to older women [8-10]. Among the five prospective studies that reported results in younger or pre-menopausal women or both, four reported no association [11-13,16]. In the largest of the four studies, the Nurses' Health Study II, which collected blood in relatively young (age range of 32 to 54 years), mostly pre-menopausal women, a large number of whom were still pre-menopausal at diagnosis (294 cases), the multivariate-adjusted OR associated with the top quintile of 25(OH)D was 1.19 (95% CI = 0.77 to 1.84, *P*_trend _= 0.51). The French E3N cohort, though, reported a significant inverse association in women who were younger (< 53 years old) at blood donation, results consistent with ours, and also observed a non-significant protective association in the smaller subgroup of women who were pre-menopausal at diagnosis. The investigators suggested that vitamin D may act by inhibiting the tumor growth-stimulating effects of IGF-1 [15]. Because IGF-1 levels decrease with age, a stronger anticarcinogenic effect of vitamin D would be expected in younger/pre-menopausal women. However, our analysis stratifying directly by IGF-1 level did not support this hypothesis, although the sample size was limited.

A protective effect of vitamin D on breast cancer would also be expected to be stronger in pre-menopausal women if vitamin D acts by inhibiting estrogen-stimulated breast cell proliferation [40], since estrogen levels are much higher before menopause. However, we found no evidence that the effect of vitamin D varies according to estrone levels in post-menopausal women, in spite of the strong positive association between estrone and breast cancer risk in our study. Moreover, 25(OH)D was not associated with either ER-positive or ER-negative breast cancer, and this is consistent with results from other prospective studies of 25(OH)D that found no difference by ER status [10,13].

Too few subjects had very high concentrations of 25(OH)D for us to be able to examine the association of concentrations of at least 100 nmol/L with breast cancer risk. However, the lack of dose response in the less than 50 to at least 75 nmol/L range (Table 4) suggests that a true association would have to be of the threshold type, a hypothesis for which there is little biological support. A linear dose-response association at levels of not more than 75 nmol/L has been observed for colorectal cancer, the one type of cancer for which there is consistent evidence of a protective effect of vitamin D [41].

Several factors that are associated with breast cancer risk are also associated with circulating 25(OH)D and therefore could confound the 25(OH)D-breast cancer association [42]. Dark skin, higher BMI, and lower physical activity have been repeatedly found to be associated with lower levels of 25(OH)D, whereas associations between 25(OH)D and current use of HRT, vitamin supplements, and alcohol have been less consistent [28,42,43]. The importance of taking into account these lifestyle factors was demonstrated in the Women's Health Initiative study, in which a significant inverse association of 25(OH)D with breast cancer risk was attenuated and became non-significant after adjustment for BMI and physical activity [42]. In our study, we matched on race/ethnicity (a surrogate for dark skin) and also conducted analyses limited to Caucasians, and this gave results similar to the analyses that included all races. As shown in Tables 3, 4, and 5, adjusting for BMI, HRT use, physical activity, and multivitamin use had very little effect on the ORs. Residual confounding is possible, particularly by physical activity, for which we classified women in one of three categories and data were missing for 11% in the NYUWHS and 38% in the NSMSC. However, an analysis limited to the subjects for whom physical activity was available showed very similar results, as did an analysis adjusting for MET-hours per week as a continuous variable in the NYUWHS (data not shown). Another potential source of confounding is breast cancer screening frequency, as screening visits that are more frequent could result in earlier diagnosis of breast cancer or correlate with other health-conscious behaviors leading to higher 25(OH)D status. However, because blood donations occurred at the time of mammographic screening visits, number of blood donations can be considered a proxy for mammographic screening frequency. We matched on number of blood donations in the NYUWHS, whereas in the NSMSC, although we did not match on this variable, 59% of cases had at least one control who matched exactly on the number of blood donations, and 89% of cases had at least one control who matched within ± 1 blood donation. We therefore believe that confounding by screening history is unlikely in our study.

We used residuals obtained by local regression to take into account seasonal variation. This method has been used only rarely in studies of 25(OH)D [44], although it has been used in epidemiologic analyses of other biomarkers with temporal variation (for instance, hormones known to vary during pregnancy) [45,46]. In our study, when the residual method was used, the exposure value for each woman was the difference between the absolute level observed for this woman and the projected mean of 25(OH)D for this day of the year (reference day). A positive residual indicated that a woman had a higher-than-average level at this time of the year, whereas a negative residual indicated a lower-than-average level. The projected mean was calculated by using all samples collected in the same cohort on the reference day, as well as samples taken on neighboring days, with progressively decreasing weights given to samples collected further away from the reference day. This method, therefore, seems well suited to take into account the gradual changes observed during the shoulder seasons, when levels progressively increase (spring) or decrease (fall).

Strengths of this study include its prospective design, inclusion of two cohorts with different diet and sun exposure, and large sample size. This is also the first study to include repeat blood samples on a large number of women. The repeat samples enabled us to assess temporal reproducibility and gave an indication of the potential impact of ignoring seasonal variation when studying the association of circulating vitamin D with disease risk. The lower ICCs observed when samples were collected in different seasons, compared with the same season, highlight the importance of taking season into account in the study design or analysis or both, as other studies have concluded [47]. The ICC of 0.63 for samples collected 8.0 to 11.7 years apart in the NSMSC compares well with the ICCs of other biomarkers that have been linked to breast cancer risk, such as post-menopausal sex hormones. However, the ICC decreased with increasing time between blood donations, although this trend did not seem to extend beyond the first 8 years. This observation underlines that a single measurement of 25(OH)D is an imperfect reflection of vitamin D status over the long time period during which breast cancer develops. Thus, the association of vitamin D status with breast cancer risk may have been underestimated because of random error in measurement of the true exposure of interest (that is, the long-term average level of 25(OH)D). Another limitation of our study is the relatively small number of subjects with very high levels of circulating 25(OH)D (≥ 100 nmol/L).

## Conclusions

This large prospective study does not support a relationship between circulating 25(OH)D and risk of breast cancer, except possibly in younger women. These results add to a growing body of evidence from prospective studies and randomized trials that suggests that higher vitamin D levels do not reduce breast cancer risk. Recommendations in regard to vitamin D supplementation should be based on considerations other than breast cancer prevention, such as bone health.

## Abbreviations

25(OH)D: 25-hydroxyvitamin D; BMI: body mass index; CI: confidence interval; CV: coefficient of variation; ER: estrogen receptor; HRT: hormone replacement therapy; ICC: intraclass correlation coefficient; IGF-I: insulin-like growth factor 1; MET: metabolic equivalent of task; MSC: Mammary Screening Cohort; NSMSC: Northern Sweden Mammary Screening Cohort; NYUWHS: New York University Women's Health Study; OR: odds ratio; VDR: vitamin D receptor.

## Competing interests

The authors declare that they have no competing interests.

## Authors' contributions

SS interpreted the data and drafted the manuscript. YA performed the statistical analysis and critically reviewed the manuscript. PL, AA, YC, GH, EL, PT, and RS critically reviewed the manuscript. KK conceived of the study design, interpreted the data, and critically reviewed the manuscript. RH performed the 25(OH)D assay and critically reviewed the manuscript. TC interpreted the data and critically reviewed the manuscript. SR performed the estrone analyses and critically reviewed the manuscript. AZ-J secured funding, conceived of the study design, interpreted the data, and drafted the manuscript. All authors read and approved the final manuscript.

## References

[B1] Welsh J Vitamin D and breast cancer: insights from animal models Am J Clin Nutr 2004 80 1721S 1724S 15585794 10.1093/ajcn/80.6.1721S

[B2] Bertone-Johnson ER Vitamin D and breast cancer Ann Epidemiol 2009 19 462 467 10.1016/j.annepidem.2009.01.003 19230714

[B3] Institute of Medicine; Food and Nutrition Board Ross AC, Taylor CL, Yaktine AL, Del Valle HB Dietary Reference Intakes for Calcium and Vitamin D 2010 Washington, DC: Institute of Medicine; Food and Nutrition Board

[B4] Friedrich M Diesing D Cordes T Fischer D Becker S Chen TC Flanagan JN Tangpricha V Gherson I Holick MF Reichrath J Analysis of 25-hydroxyvitamin D3-1alpha-hydroxylase in normal and malignant breast tissue Anticancer Res 2006 26 2615 2620 16886671

[B5] Davis CD Vitamin D and cancer: current dilemmas and future research needs Am J Clin Nutr 2008 88 565S 569S 18689403 10.1093/ajcn/88.2.565S

[B6] Prentice A Goldberg GR Schoenmakers I Vitamin D across the lifecycle: physiology and biomarkers Am J Clin Nutr 2008 88 500S 506S 18689390 10.1093/ajcn/88.2.500S

[B7] Welsh J Wietzke JA Zinser GM Byrne B Smith K Narvaez CJ Vitamin D-3 receptor as a target for breast cancer prevention J Nutr 2003 133 2425S 2433S 12840219 10.1093/jn/133.7.2425S

[B8] Chlebowski RT Johnson KC Kooperberg C Pettinger M Wactawski-Wende J Rohan T Rossouw J Lane D O'Sullivan MJ Yasmeen S Hiatt RA Shikany JM Vitolins M Khandekar J Hubbell FA Women's Health Initiative Investigators Calcium plus vitamin d supplementation and the risk of breast cancer J Natl Cancer Inst 2008 100 1581 1591 10.1093/jnci/djn360 19001601 PMC2673920

[B9] Freedman DM Chang SC Falk RT Purdue MP Huang WY McCarty CA Hollis BW Graubard BI Berg CD Ziegler RG Serum levels of vitamin D metabolites and breast cancer risk in the prostate, lung, colorectal, and ovarian cancer screening trial Cancer Epidemiol Biomarkers Prev 2008 17 889 894 10.1158/1055-9965.EPI-07-2594 18381472 PMC4039037

[B10] McCullough ML Stevens VL Patel R Jacobs EJ Bain EB Horst RL Gapstur SM Thun MJ Calle EE Serum 25-hydroxyvitamin D concentrations and postmenopausal breast cancer risk: a nested case control study in the Cancer Prevention Study-II Nutrition Cohort Breast Cancer Res 2009 11 R64 10.1186/bcr2356 19715600 PMC2750126

[B11] Agborsangaya CB Surcel HM Toriola AT Pukkala E Parkkila S Tuohimaa P Lukanova A Lehtinen M Serum 25-hydroxyvitamin D at pregnancy and risk of breast cancer in a prospective study Eur J Cancer 2010 46 467 470 10.1016/j.ejca.2009.11.019 20022237

[B12] Almquist M Bondeson AG Bondeson L Malm J Manjer J Serum levels of vitamin D, PTH and calcium and breast cancer risk-a prospective nested case-control study Int J Cancer 2010 127 2159 2168 10.1002/ijc.25215 20112341

[B13] Eliassen AH Spiegelman D Hollis BW Horst RL Willett WC Hankinson SE Plasma 25-hydroxyvitamin D and risk of breast cancer in the Nurses' Health Study II Breast Cancer Res 2011 13 R50 10.1186/bcr2880 21569367 PMC3218936

[B14] Amir E Cecchini RS Ganz PA Costantino JP Beddows S Hood N Goodwin PJ 25-Hydroxy vitamin-D, obesity, and associated variables as predictors of breast cancer risk and tamoxifen benefit in NSABP-P1 Breast Cancer Res Treat 2012 133 1077 1088 10.1007/s10549-012-2012-x 22415479 PMC3396331

[B15] Engel P Fagherazzi G Boutten A Dupre T Mesrine S Boutron-Ruault MC Clavel-Chapelon F Serum 25(OH) vitamin D and risk of breast cancer: a nested case-control study from the French E3N cohort Cancer Epidemiol Biomarkers Prev 2010 19 2341 2350 10.1158/1055-9965.EPI-10-0264 20826834

[B16] Bertone-Johnson ER Chen WY Holick MF Hollis BW Colditz GA Willett WC Hankinson SE Plasma 25-hydroxyvitamin D and 1,25-dihydroxyvitamin D and risk of breast cancer Cancer Epidemiol Biomarkers Prev 2005 14 1991 1997 10.1158/1055-9965.EPI-04-0722 16103450

[B17] Rejnmark L Tietze A Vestergaard P Buhl L Lehbrink M Heickendorff L Mosekilde L Reduced prediagnostic 25-hydroxyvitamin D levels in women with breast cancer: a nested case-control study Cancer Epidemiol Biomarkers Prev 2009 18 2655 2660 10.1158/1055-9965.EPI-09-0531 19789365

[B18] Chung M Lee J Terasawa T Lau J Trikalinos TA Vitamin D with or without calcium supplementation for prevention of cancer and fractures: an updated meta-analysis for the U.S. Preventive Services Task Force Ann Intern Med 2011 155 827 838 22184690 10.7326/0003-4819-155-12-201112200-00005

[B19] International Agency for Research on Cancer Vitamin D and cancer IARC Working Group Reports 2008 5 Lyon, France: International Agency for Research on Cancer 143 148

[B20] Toniolo PG Pasternack BS Shore RE Sonnenschein E Koenig KL Rosenberg C Strax P Strax S Endogenous hormones and breast cancer: a prospective cohort study Breast Cancer Res Treat 1991 18 Suppl 1 S23 26 1873553 10.1007/BF02633522

[B21] Hallmans G Agren A Johansson G Johansson A Stegmayr B Jansson JH Lindahl B Rolandsson O Söderberg S Nilsson M Johansson I Weinehall L Cardiovascular disease and diabetes in the Northern Sweden Health and Disease Study Cohort - evaluation of risk factors and their interactions Scand J Public Health Suppl 2003 61 18 24 14660243 10.1080/14034950310001432

[B22] Kato I Toniolo P Koenig KL Kahn A Schymura M Zeleniuch-Jacquotte A Comparison of active and cancer registry-based follow-up for breast cancer in a prospective cohort study Am J Epidemiol 1999 149 372 378 10.1093/oxfordjournals.aje.a009823 10025481

[B23] Wagner D Hanwell HE Vieth R An evaluation of automated methods for measurement of serum 25-hydroxyvitamin D Clin Biochem 2009 42 1549 1556 10.1016/j.clinbiochem.2009.07.013 19631201

[B24] Zeleniuch-Jacquotte A Shore RE Koenig KL Akhmedkhanov A Afanasyeva Y Kato I Kim MY Rinaldi S Kaaks R Toniolo P Postmenopausal levels of oestrogen, androgen, and SHBG and breast cancer: long-term results of a prospective study Br J Cancer 2004 90 153 159 10.1038/sj.bjc.6601517 14710223 PMC2395327

[B25] Rinaldi S Déchaud H Biessy C Morin-Raverot V Toniolo P Zeleniuch-Jacquotte A Akhmedkhanov A Shore RE Secreto G Ciampi A Riboli E Kaaks R Reliability and validity of commercially available, direct radioimmunoassays for measurement of blood androgens and estrogens in postmenopausal women Cancer Epidemiol Biomarkers Prev 2001 10 757 765 11440961

[B26] Borkowf CB Albert PS Abnet CC Using lowess to remove systematic trends over time in predictor variables prior to logistic regression with quantile categories Stat Med 2003 22 1477 1493 10.1002/sim.1507 12704611

[B27] Brock K Huang WY Fraser DR Ke L Tseng M Stolzenberg-Solomon R Peters U Ahn J Purdue M Mason RS McCarty C Ziegler RG Graubard B Low vitamin D status is associated with physical inactivity, obesity and low vitamin D intake in a large US sample of healthy middle-aged men and women J Steroid Biochem Mol Biol 2010 121 462 466 10.1016/j.jsbmb.2010.03.091 20399270 PMC2906665

[B28] McCullough ML Weinstein SJ Freedman DM Helzlsouer K Flanders WD Koenig K Kolonel L Laden F Le Marchand L Purdue M Snyder K Stevens VL Stolzenberg-Solomon R Virtamo J Yang G Yu K Zheng W Albanes D Ashby J Bertrand K Cai H Chen Y Gallicchio L Giovannucci E Jacobs EJ Hankinson SE Hartge P Hartmuller V Harvey C Hayes RB Correlates of circulating 25-hydroxyvitamin D: cohort consortium vitamin D pooling project of rarer cancers Am J Epidemiol 2010 172 21 35 10.1093/aje/kwq113 20562191 PMC2892536

[B29] Winzer BM Whiteman DC Reeves MM Paratz JD Physical activity and cancer prevention: a systematic review of clinical trials Cancer Causes Control 2011 22 811 826 10.1007/s10552-011-9761-4 21461921

[B30] van Buuren S Multiple imputation of discrete and continuous data by fully conditional specification Stat Methods Med Res 2007 16 219 242 10.1177/0962280206074463 17621469

[B31] Chen P Hu P Xie D Qin Y Wang F Wang H Meta-analysis of vitamin D, calcium and the prevention of breast cancer Breast Cancer Res Treat 2010 121 469 477 10.1007/s10549-009-0593-9 19851861

[B32] Chlebowski RT Vitamin D and breast cancer: interpreting current evidence Breast Cancer Res 2011 13 217 10.1186/bcr2846 21884640 PMC3236325

[B33] Janowsky EC Lester GE Weinberg CR Association between low levels of 1,25-dihydroxyvitamin D and breast cancer risk Public Health Nutr 1999 2 283 291 10512563 10.1017/s1368980099000385

[B34] Abbas S Linseisen J Slanger T Kropp S Mutschelknauss E Flesch-Janys D Chang-Claude J Serum 25-hydroxyvitamin D and risk of post-menopausal breast cancer--results of a large case-control study Carcinogenesis 2008 29 93 99 17974532 10.1093/carcin/bgm240

[B35] Abbas S Chang-Claude J Linseisen J Plasma 25-hydroxyvitamin D and premenopausal breast cancer risk in a German case-control study Int J Cancer 2009 124 250 255 10.1002/ijc.23904 18839430

[B36] Crew KD Gammon MD Steck SE Hershman DL Cremers S Dworakowski E Shane E Terry MB Desai M Teitelbaum SL Neugut AI Santella RM Association between plasma 25-hydroxyvitamin D and breast cancer risk Cancer Prev Res (Phila) 2009 2 598 604 10.1158/1940-6207.CAPR-08-0138 19470790 PMC3077714

[B37] Lowe LC Guy M Mansi JL Peckitt C Bliss J Wilson RG Colston KW Plasma 25-hydroxy vitamin D concentrations, vitamin D receptor genotype and breast cancer risk in a UK Caucasian population Eur J Cancer 2005 41 1164 1169 10.1016/j.ejca.2005.01.017 15911240

[B38] Dorgan J Stanczyk F Kahle L Brinton L Prospective case-control study of premenopausal serum estradiol and testosterone levels and breast cancer risk Breast Cancer Res 2010 12 R98 10.1186/bcr2779 21087481 PMC3046441

[B39] Fedirko V Torres-Mejía G Ortega-Olvera C Biessy C Angeles-Llerenas A Lazcano-Ponce E Saldaña-Quiroz VA Romieu I Serum 25-hydroxyvitamin D and risk of breast cancer: results of a large population-based case-control study in Mexican women Cancer Causes Control 2012 23 1149 1162 10.1007/s10552-012-9984-z 22622862

[B40] Welsh J Wietzke J Zinser G Smyczek S Romu S Tribble E Welsh J Byrne B Narvaez C Impact of the Vitamin D3 receptor on growth-regulatory pathways in mammary gland and breast cancer J Steroid Biochem Mol Biol 2002 83 85 92 10.1016/S0960-0760(02)00277-7 12650704

[B41] Zhang X Giovannucci E Calcium, vitamin D and colorectal cancer chemoprevention Best Pract Res Clin Gastroenterol 2011 25 485 494 10.1016/j.bpg.2011.10.001 22122765

[B42] Neuhouser ML Manson JE Millen A Pettinger M Margolis K Jacobs ET Shikany JM Vitolins M Adams-Campbell L Liu S LeBlanc E Johnson KC Wactawski-Wende J The influence of health and lifestyle characteristics on the relation of serum 25-hydroxyvitamin D with risk of colorectal and breast cancer in postmenopausal women Am J Epidemiol 2012 175 673 684 10.1093/aje/kwr350 22362582 PMC3324430

[B43] Millen AE Wactawski-Wende J Pettinger M Melamed ML Tylavsky FA Liu S Robbins J LaCroix AZ LeBoff MS Jackson RD Predictors of serum 25-hydroxyvitamin D concentrations among postmenopausal women: the Women's Health Initiative Calcium plus Vitamin D clinical trial Am J Clin Nutr 2010 91 1324 1335 10.3945/ajcn.2009.28908 20219959 PMC2854906

[B44] Gallicchio L Helzlsouer KJ Chow WH Freedman DM Hankinson SE Hartge P Hartmuller V Harvey C Hayes RB Horst RL Koenig KL Kolonel LN Laden F McCullough ML Parisi D Purdue MP Shu XO Snyder K Stolzenberg-Solomon RZ Tworoger SS Varanasi A Virtamo J Wilkens LR Xiang YB Yu K Zeleniuch-Jacquotte A Zheng W Abnet CC Albanes D Circulating 25-hydroxyvitamin D and the risk of rarer cancers: design and methods of the cohort consortium vitamin D pooling project of rarer cancers Am J Epidemiol 2010 172 10 20 10.1093/aje/kwq116 20562188 PMC2892539

[B45] Lukanova A Andersson R Wulff M Zeleniuch-Jacquotte A Grankvist K Dossus L Afanasyeva Y Johansson R Arslan AA Lenner P Wadell G Hallmans G Toniolo P Lundin E Human chorionic gonadotropin and alpha-fetoprotein concentrations in pregnancy and maternal risk of breast cancer: a nested case-control study Am J Epidemiol 2008 168 1284 1291 10.1093/aje/kwn254 18936438 PMC2587527

[B46] Richardson BE Hulka BS David Peck JL Hughes CL van den Berg BJ Christianson RE Calvin JA Levels of maternal serum alpha-fetoprotein (AFP) in pregnant women and subsequent breast cancer risk Am J Epidemiol 1998 148 719 727 10.1093/oxfordjournals.aje.a009691 9786226

[B47] Jorde R Sneve M Hutchinson M Emaus N Figenschau Y Grimnes G Tracking of serum 25-hydroxyvitamin D levels during 14 years in a population-based study and during 12 months in an intervention study Am J Epidemiol 2010 171 903 908 10.1093/aje/kwq005 20219763

